# Supplementary material for: Identifying multivariate disease trajectories and potential phenotypes of early knee osteoarthritis in the CHECK cohort
Source: PLoS One. 2023 Jul 14;18(7):e0283717. doi: 10.1371/journal.pone.0283717 (PMC10348540; doi:10.1371/journal.pone.0283717)
Supplement: S1 Table — *We removed records of knees with missing data for three or more time points and used linear interpolation to input remaining missing values. (DOCX) [file pone.0283717.s001.docx]

Supplementary Table 1: Final list of variables included in the functional analysis.

| **Parameter** | **Scale** | **Years available** | **Records after**  **Imputation*** |
| --- | --- | --- | --- |
| **Questionnaire data: WOMAC - at subject level** | | | |
| WOMAC Pain: standardized pain scale | 0-100 | Yearly (0-10) | 819 |
| WOMAC Function: standardized physical functioning scale | 0-100 | Yearly (0-10) | 819 |
| WOMAC Stiffness: standardized stiffness scale | 0-100 | Yearly (0-10) | 820 |
| **Radiographic variables: Tibiofemoral Knee Images Digital Analysis (KIDA) - at knee level** | | | |
| Lateral Osteophytes: mean of femur and area | ≥0 in mm^2^ | 0,2,5,8,10 | 1888 |
| Medial Osteophytes: mean of femur and tibia area | ≥0 in mm^2^ | 0,2,5,8,10 | 1888 |
| Lateral Joint Space Width (mean) | ≥0 in mm | 0,2,5,8,10 | 1889 |
| Medial Joint Space Width (mean) | ≥0 in mm | 0,2,5,8,10 | 1888 |
| Lateral Bone Density: mean of femur and tibia area | ≥0 in mmAl | 0,2,5,8,10 | 1846 |
| Medial Bone Density: mean of femur and tibia area | ≥0 in mmAl | 0,2,5,8,10 | 1846 |
|  |  | *Combined:* | 1788 |
| **Radiographic variables: Patellofemoral OA Scoring (skyline views) - at knee level** | | | |
| Knee sky patellofemoral sclerosis | 0,1,2,3 | 0,2,5,8,10 | 1788 |
| Knee sky patellofemoral narrowing | 0,1,2,3 | 0,2,5,8,10 | 1877 |
| Knee sky patellofemoral osteophytes | 0,1,2,3 | 0,2,5,8,10 | 1873 |
|  |  | *Combined:* | 1788 |

*We removed records of knees with missing data for three or more time points and used linear interpolation to input remaining missing values.
