# Supplementary material for: Identifying multivariate disease trajectories and potential phenotypes of early knee osteoarthritis in the CHECK cohort
Source: PLoS One. 2023 Jul 14;18(7):e0283717. doi: 10.1371/journal.pone.0283717 (PMC10348540; doi:10.1371/journal.pone.0283717)
Supplement: S2 Table — ROM: range of motion; CTX-I: C-terminal telopeptide of collagen I; CTX-II: C-terminal telopeptide of type II collagen; C1,2C: collagen of types I and II; COMP: cartilage oligomeric matrix protein; PIIANP: collagen N-propeptide of type IIA; CS846: chondroitin sulphate 846; NTX-I: N-terminal telopeptide of collagen I; OC: osteocalcin; PINP: aminoterminal propeptide of type I procollagen; HA: hyaluronic acid; PIIIANP: N-terminal propeptide of type III procollagen; hsCRP: high-sensitivity C-reactive protein; BSE: erythrocyte sedimentation rate. * P-values calculated with chi-square test. **for missing values, mean of all SOS items was taken (n = 6). Median and interquartile range (Q1-Q3) given for continuous variables unless otherwise indicated. (DOCX) [file pone.0283717.s002.docx]

Supplementary Table 2: Descriptive statistics of combined scenario with WOMAC and PF OA features

| **Characteristics** | **Cluster 1** | **Cluster 2** | **Cluster 3** | **Cluster 4** | **Cluster 5** | **Cluster 6** | **P-value** |
| --- | --- | --- | --- | --- | --- | --- | --- |
|  | low PF JSN, low WOMAC pain and function | low WOMAC pain and function | high WOMAC pain and function | moderate WOMAC features, low JSN | moderate WOMAC features | increasing then stable osteophytes, low JSN, moderate WOMAC features |  |
| n=1605 | n=183 (11%) | n=349 (22%) | **n=137 (9%)** | n=327 (20%) | n=372 (23%) | n=237 (15%) |  |
| Age, years | 57.0 (52.0-60.0) | 57.0 (52.0-60.0) | 56.0 (52.0-61.0) | 56.0 (52.0-60.0) | 55.0 (52.0-60.0) | 56.0 (52.0-60.0) | 0.53 |
| Female, n (%) | 139 (76.0) | 283 (81.1) | 113 (82.5) | 254 (77.7) | 292 (78.5) | 182 (76.8) | 0.56* |
| Body Mass Index, kg/m^2^ | 25.0 (23.0-27.0) | 26.0 (24.0-29.0) | 25.0 (23.0-28.0) | 25.0 (23.0-28.0) | 25.5 (23.0-28.0) | 25.0 (23.0-29.0) | 0.18 |
| Highest completed education level, n (%)   - No school/primary school - Basic vocational education - Secondary education - Secondary vocational education - Higher and university preparatory education - Higher professional education - University | 2 (1.1)  33 (18.6)  41 (23.2)  32 (18.1)  10 (5.6)  46 (26.0)  13 (7.3) | 3 (0.9)  53 (15.8)  82 (24.4)  59 (17.6)  24 (7.1)  88 (26.2)  27 (8.0) | 4 (3.0)  24 (18.0)  32 (24.1)  22 (16.5)  15 (11.3)  29 (21.8)  7 (5.3) | 10 (3.2)  55 (17.4)  83 (26.3)  67 (21.2)  27 (8.5)  57 (18.0)  17 (5.4) | 12 (3.3)  54 (14.8)  105 (28.8)  86 (23.6)  24 (6.6)  59 (16.2)  24 (6.6) | 9 (3.8)  41 (17.4)  60 (25.5)  47 (20.0)  18 (7.7)  44 (18.7)  16 (6.8) | 0.30* |
| Smoking, n (%)   - Yes, every day - Yes, occasionally - No, but used to every day - No, but used to occasionally - No, never have | 16 (8.9)  5 (2.8)  59 (33.0)  48 (26.8)  51 (28.5) | 38 (11.2)  13 (3.8)  105 (31.0)  75 (22.1)  108 (31.9) | 4 (2.9)  3 (2.2)  44 (32.1)  32 (23.4)  54 (39.4) | 26 (8.2)  15 (4.7)  114 (36.1)  65 (20.6)  90 (30.4) | 40 (11.0)  30 (8.2)  124 (34.1)  75 (20.6)  95 (26.1) | 17 (7.3)  13 (5.6)  74 (31.6)  48 (20.5)  82 (35.0) | 0.02* |
| Number of comorbidities | 1.0 (0.0-3.0) | 1.0 (0.0-2.0) | 1.0 (0.5-2.0) | 1.0 (0.0-2.0) | 1.0 (0.0-2.0) | 1.0 (1.0-3.0) | 0.76 |
| Social Support Scale (SOS) total score** | 16.0 (12.0-20.0) | 15.0 (12.0-20.0) | 14.0 (12.0-22.0) | 16.0 (12.0-22.0) | 15.0 (12.0-20.0) | 15.0 (12.0-20.0) | 0.55 |
| Pain Coping Inventory transformation | 2.0 (1.5-2.5) | 2.0 (1.8-2.7) | 2.0 (1.8-2.5) | 2.0 (1.7-2.5) | 2.0 (1.8-2.5) | 2.3 (1.8-2.8) | 0.06 |
| Pain Coping Inventory distraction | 2.2 (1.8-2.6) | 2.2 (1.8-2.6) | 2.2 (1.8-2.7) | 2.2 (1.8-2.6) | 2.2 (1.6-2.6) | 2.4 (1.8-2.8) | 0.40 |
| Pain Coping Inventory reducing demands | 2.0 (1.7-2.3) | 2.0 (1.7-2.3) | 2.0 (1.7-2.7) | 2.0 (1.7-2.3) | 2.0 (1.7-2.3) | 2.0 (1.7-2.3) | 0.75 |
| Pain Coping Inventory retreating | 1.4 (1.1-2.0) | 1.6 (1.1-2.0) | 1.5 (1.1-1.7) | 1.4 (1.1-1.9) | 1.4 (1.1-1.9) | 1.6 (1.1-1.9) | 0.07 |
| Pain Coping Inventory worrying | 1.4 (1.2-1.8) | 1.4 (1.2-1.8) | 1.4 (1.3-1.9) | 1.4 (1.2-1.8) | 1.4 (1.2-1.8) | 1.6 (1.2-1.9) | 0.11 |
| Pain Coping Inventory resting | 1.8 (1.4-2.2) | 1.8 (1.5-2.2) | 1.8 (1.6-2.4) | 1.8 (1.4-2.2) | 1.8 (1.4-2.2) | 1.8 (1.4-2.2) | 0.07 |
| Knee flexion active ROM, degrees | 135.0 (130.0-140.0) | 136.0 (130.0-142.0) | 135.0 (130.0-140.0) | 137.0 (130.0-140.0) | 135.0 (130.0-141.0) | 135.0 (130.0-140.3) | 0.64 |
| Knee extension active ROM, degrees | 2.0 (0.0-5.0) | 2.0 (0.0-5.0) | 0.0 (0.0-5.0) | 2.0 (0.0-5.0) | 2.0 (0.0-5.0) | 2.0 (0.0-5.0) | 0.33 |
| Hip endorotation active ROM, degrees | 30.0 (23.0-35.0) | 30.0 (25.0-35.0) | 30.0 (25.0-37.0) | 30.0 (25.0-37.0) | 30.0 (25.0-35.0) | 30.0 (25.0-38.0) | 0.02 |
| Hip exorotation active ROM, degrees | 27.0 (21.0-35.0) | 29.5 (22.0-35.0) | 30.0 (25.0-35.0) | 27.0 (21.5-35.0) | 30.0 (24.0-35.0) | 28.0 (22.0-34.5) | 0.04 |
| Hip flexion active ROM, degrees | 119.0 (110.0-125.0) | 120.0 (110.0-125.0) | 120.0 (110.0-125.0) | 120.0 (110.0-126.0) | 120.0 (110.0-125.0) | 120.0 (110.0-130.0) | 0.75 |
| Hip adduction active ROM, degrees | 20.0 (15.0-25.0) | 20.0 (15.0-25.0) | 20.0 (15.3-29.8) | 20.0 (15.0-24.5) | 20.0 (15.5-25.5) | 20.0 (15.0-25.0) | 0.34 |
| Hip abduction active ROM, degrees | 30.0 (20.0-40.0) | 30.0 (25.0-40.0) | 34.0 (27.0-40.0) | 30.0 (25.0-40.0) | 31.0 (25.0-40.0) | 30.0 (25.0-40.0) | 0.28 |
| Kellgren & Lawrence grade, n (%)   - Grade 0 - Grade 1 - Grade 2 - Grade 3 | 118 (65.6)  44 (24.4)  18 (10.0)  0 (0.0) | 198 (60.4)  98 (29.9)  32 (9.8)  0 (0.0) | 79 (59.4)  39 (29.3)  15 (11.3)  0 (0.0) | 183 (58.1)  103 (32.7)  29 (9.2)  0 (0.0) | 222 (63.1)  87 (24.7)  43 (12.2)  0 (0.0) | 140 (61.1)  62 (27.1)  26 (11.4)  1 (0.4) | 0.514* |
| Pain or stiffness in hip and/or groin and/or upper part of the upper leg, n (%) | 70 (39.1) | 142 (41.9) | 54 (39.4) | 115 (36.2) | 151 (41.1) | 96 (40.9) | 0.73* |
| Pain or stiffness in knee, n (%) | 104 (58.1) | 199 (58.7) | 78 (56.9) | 188 (59.1) | 226 (61.6) | 130 (55.3) | 0.76* |
| pLeptin, ng/ml | 10.3 (5.5-18.3) | 12.9 (6.1-24.1) | 11.5 (6.4-19.3) | 11.0 (6.7-19.6) | 11.8 (6.3-23.8) | 10.9 (5.5-25.2) | 0.35 |
| pAdiponectin, ug/ml | 9.4 (7.4-13.5) | 10.0 (7.5-14.6) | 11.0 (7.9-16.7) | 9.7 (7.1-14.7) | 9.6 (6.9-14.0) | 9.6 (7.1-14.4) | 0.44 |
| pResistin, ng/ml | 3.5 (3.0-4.3) | 3.6 (3.0-4.4) | 3.0 (4.2) | 3.4 (2.9-4.2) | 3.5 (2.9-4.2) | 3.5 (2.9-4.3) | 0.50 |
| uCTX-I, ug/mmol | 144.4 (88.5-220.4) | 157.0 (100.9-223.7) | 178.8 (114.7-245.3) | 152.5 (103.2-224.6) | 103.8 (153.6-236.0) | 153.6 (105.6-221.2) | 0.38 |
| uNTX-I, nM BCE/mmol | 35.1 (26.1-49.3) | 36.2 (27.7-49.9) | 40.8 (30.2-50.6) | 36.9 (28.4-51.3) | 37.5 (26.8-51.2) | 36.6 (29.7-51.0) | 0.29 |
| sPINP, ug/ml | 39.3 (29.9-55.9) | 42.4 (30.4-55.9) | 46.6 (34.3-59.8) | 41.5 (32.7-54.5) | 43.2 (33.2-56.1) | 41.9 (32.8-53.5) | 0.27 |
| sOC | 12.7 (10.1-17.3) | 13.2 (10.7-16.4) | 14.3 (11.0-17.1) | 12.7 (10.5-16.0) | 13.3 (10.6-17.1) | 13.3 (10.7-16.8) | 0.62 |
| sC1,2C | 0.18 (0.14-0.23) | 0.18 (0.14-0.22) | 0.16 (0.13-0.22) | 0.17 (0.14-0.22) | 0.18 (0.14-0.22) | 0.16 (0.13-0.21) | 0.10 |
| uCTX-II, ng/mmol | 188.5 (136.9-281.2) | 190.0 (134.1-285.7) | 167.3 (126.1-296.4) | 191.1 (128.3-293.1) | 195.0 (127.9-283.2) | 211.7 (143.8-268.0) | 0.82 |
| sCS846 | 68.8 (52.4-85.7) | 71.2 (56.3-88.6) | 75.0 (55.6-90.6) | 68.6 (53.6-87.7) | 70.2 (53.0-86.7) | 71.1 (56.1-90.7) | 0.48 |
| sCOMP, μg/ml | 8.3 (7.3-10.0) | 8.4 (7.2-10.0) | 8.5 (7.0-9.8) | 8.7 (7.3-10.1) | 8.5 (7.2-10.2) | 8.5 (7.0-9.7) | 0.55 |
| sPIIANP | 1353.4 (1020.8-1764.8) | 1391.0 (1096.2-1760.1) | 1474.1 (1154.5-1831.9) | 1395.4 (1090.1-1721.5) | 1381.2 (1056.6-1763.9) | 1431.1 (1111.4-1818.0) | 0.49 |
| sHA | 26.2 (16.7-45.6) | 27.7 (18.8-45.2) | 27.0 (15.2-44.3) | 28.0 (17.4-41.9) | 26.0 (16.7-43.9) | 26.6 (16.2-42.2) | 0.92 |
| sPIIIANP | 4.2 (3.6-4.8) | 4.1 (3.6-5.0) | 4.3 (3.5-5.3) | 4.0 (3.6-5.0) | 4.1 (3.6-4.9) | 4.1 (3.5-4.8) | 0.88 |
| hsCRP | 1.2 (0.5-2.7) | 1.6 (0.8-3.0) | 1.3 (0.6-2.6) | 0.7 (1.3-3.3) | 1.6 (0.7-3.6) | 1.3 (0.7-3.7) | 0.23 |
| BSE | 8.0 (5.0-13.0) | 8.0 (5.0-13.0) | 8.0 (5.0-14.5) | 7.0 (5.0-13.0) | 8.0 (5.0-13.0) | 8.0 (5.0-13.0) | 0.78 |
| WOMAC pain scale standardized (0-100) | 25.0 (15.0-40.0) | 25.0 (10.0-35.0) | 20.0 (12.5-35.0) | 25.0 (10.0-35.0) | 25.0 (10.0-35.0) | 25.0 (10.0-35.0) | 0.59 |
| WOMAC physical functioning scale standardized (0-100) | 23.5 (10.3-35.3) | 20.6 (10.3-35.3) | 16.2 (8.8-30.9) | 19.1 (10.3-32.4) | 19.1 (8.8-33.8) | 20.6 (11.0-35.3) | 0.22 |
| WOMAC stiffness scale standardized (0-100) | 37.5 (12.5-50.0) | 37.5 (12.5-50.0) | 25.0 (12.5-50.0) | 25.0 (25.0-50.0) | 37.5 (25.0-50.0) | 37.5 (12.5-50.0) | 0.85 |
| Lateral Osteophytes: mean of femur and tibia area, mm^2^ | 1.2 (0.0-2.7) | 1.3 (0.0-2.8) | 1.4 (0.0-3.1) | 1.6 (0.0-3.2) | 1.1 (0.0-2.9) | 1.1 (0.0-2.5) | 0.21 |
| Medial Osteophytes: mean of femur and tibia area, mm^2^ | 0.8 (0.0-1.6) | 0.7 (0.0-2.1) | 0.7 (0.0-1.8) | 0.7 (0.0-2.0) | 0.6 (0.0-1.9) | 0.5 (0.0-1.7) | 0.69 |
| Lateral Joint Space Width, mm | 6.1 (5.3-7.0) | 6.1 (5.2-7.1) | 6.0 (5.1-6.9) | 5.9 (5.0-6.9) | 5.9 (5.2-7.0) | 5.7 (5.0-6.7) | 0.30 |
| Medial Joint Space Width, mm | 4.7 (4.1-5.2) | 4.6 (4.1-5.2) | 4.7 (4.1-5.2) | 4.7 (4.1-5.3) | 4.7 (4.1-5.2) | 4.7 (4.0-5.3) | 0.99 |
| Lateral Bone Density: mean of femur and tibia area, mm Al eq | 23.0 (20.2-26.6) | 24.1 (21.1-28.9) | 23.4 (20.3-27.2) | 25.7 (21.5-30.1) | 24.6 (20.6-29.7) | 23.3 (20.0-26.7) | 0.00 |
| Medial Bone Density: mean of femur and tibia area, mm Al eq | 22.4 (18.6-27.2) | 23.9 (20.4-29.6) | 22.8 (18.0-28.7) | 25.3 (19.7-30.8) | 24.7 (20.0-30.4) | 22.8 (19.3-28.4) | 0.00 |
| Knee sky patellofemoral sclerosis score, n (%)   - 0 - 1 - 2 - 3 | 169 (98.8)  2 (1.2)  0 (0.0)  0 (0.0) | 293 (99.7)  0 (0.0)  1 (0.3)  0 (0.0) | 113 (98.3)  2 (1.7)  0 (0.0)  0 (0.0) | 282 (98.3)  3 (1.0)  2 (0.7)  0 (0.0) | 302 (98.1)  5 (1.6)  1 (0.3)  0 (0.0) | 202 (99.0)  2 (1.0)  0 (0.0)  0 (0.0) | 0.64* |
| Knee sky patellofemoral narrowing, n (%)   - 0 - 1 - 2 - 3 | 153 (86.0)  21 (11.8)  3 (1.7)  3 (0.6) | 287 (88.3)  34 (10.5)  4 (1.2)  0 (0.0) | 114 (88.4)  12 (9.3)  2 (1.6)  1 (0.8) | 271 (86.9)  34 (10.9)  7 (2.2)  0 (0.0) | 285 (83.1)  46 (13.4)  8 (2.3)  4 (1.2) | 187 (83.9)  31 (13.9)  5 (2.2)  0 (0.0) | 0.50* |
| Knee sky patellofemoral osteophytes, n (%)   - 0 - 1 - 2 - 3 | 85 (48.0)  74 (41.8)  16 (9.0)  2 (1.1) | 152 (47.6)  130 (40.8)  37 (11.6)  0 (0.0) | 57 (44.9)  52 (40.9)  15 (11.8)  3 (2.4) | 148 (48.1)  116 (37.7)  39 (12.7)  5 (1.6) | 161 (47.5)  132 (38.9)  39 (11.5)  7 (2.1) | 111 (50.5)  86 (39.1)  23 (10.5)  0 (0.0) | 0.54* |
| Knee replacement, n (%) | 2 (1.1) | 10 (2.9) | 0 (0.0) | 9 (2.8) | 15 (4.0) | 9 (3.8) | 0.12 |
| Hip replacement, n (%) | 11 (6.0) | 12 (3.4) | 3 (2.2) | 17 (5.2) | 17 (4.6) | 17 (7.2) | 0.21 |
| Membership to WOMAC pain trajectories, n (%)   - Moderate - Increasing - Decreasing - High - Low | 10 (5.5)  5 (2.7)  0 (0.0)  0 (0.0)  168 (91.8) | 10 (2.9)  5 (1.4)  5 (1.4)  0 (0.0)  329 (94.3) | 2 (1.5)  2 (1.5)  0 (0.0)  133 (97.1)  0 (0.0) | 238 (72.8)  7 (2.1)  55 (16.8)  4 (1.2)  23 (7.0) | 273 (73.4)  16 (4.3)  23 (6.2)  9 (2.4)  51 (13.7) | 89 (37.6)  86 (36.3)  10 (4.2)  5 (2.1)  47 (19.8) | 0.00* |
| Membership to WOMAC function trajectories, n (%)   - Moderate - Increasing - Decreasing - High - Low | 25 (13.7)  1 (0.5)  2 (1.1)  0 (0.0)  155 (84.7) | 34 (9.7)  0 (0.0)  1 (0.3)  0 (0.0)  314 (90.0) | 17 (12.5)  0 (0.0)  20 (14.6)  100 (73.0)  0 (0.0) | 139 (42.5)  7 (2.1)  113 (34.6)  35 (10.7)  33 (10.1) | 271 (72.8)  6 (1.6)  58 (15.6)  22 (5.9)  15 (4.0) | 142 (59.9)  57 (24.1)  8 (3.4)  3 (1.3)  27 (11.4) | 0.07* |
| Membership to WOMAC stiffness trajectories, n (%)   - Moderate - Increasing - Decreasing - High - Low | 69 (37.7)  25 (13.7)  28 (15.3)  29 (15.8)  32 (17.5) | 140 (40.1)  36 (10.3)  37 (10.6)  54 (15.5)  82 (23.5) | 54 (39.4)  10 (7.3)  15 (10.9)  36 (19.0)  32 (23.4) | 107 (32.7)  46 (14.1)  33 (10.1)  48 (14.7)  93 (28.4) | 156 (41.9)  38 (10.2)  41 (11.0)  63 (16.9)  74 (19.9) | 102 (43.0)  19 (8.0)  22 (9.3)  30 (12.7)  64 (27.0) | 0.07* |
| Membership to TF OA trajectories, n (%)   - Low osteophytes, stable bone density - Increasing bone density - Low bone density - Increasing lateral osteophytes, average bone density - Increasing (lateral and medial) osteophytes, low medial JSW, high lateral JSW, increasing bone density - Moderate high bone density - Increasing high bone density - Slightly increasing bone density | 41 (22.4)  28 (15.3)  13 (7.1)  19 (10.4)  8 (4.4)  41 (22.4)  16 (8.7)  17 (9.3) | 82 (23.5)  62 (17.8)  41 (11.7)  27 (7.7)  17 (4.9)  67 (19.2)  17 (4.9)  36 (10.3) | 29 (21.2)  20 (14.6)  12 (8.8)  5 (3.6)  6 (4.4)  30 (21.9)  12 (8.8)  23 (16.8) | 66 (20.2)  51 (15.6)  34 (10.4)  26 (8.0)  8 (2.4)  87 (26.6)  19 (5.8)  36 (11.0) | 72 (19.4)  61 (16.4)  45 (12.1)  33 (8.9)  11 (3.0)  88 (23.7)  18 (4.8)  44 (11.8) | 55 (23.2)  30 (12.7)  39 (16.5)  14 (5.9)  8 (3.4)  47 (19.8)  9 (3.8)  35 (14.8) | 0.15* |
| Membership to PF OA trajectories, n (%)   - Low joint space narrowing, moderate osteophytes - Moderate-increasing OA features - Low OA features - Low joint space narrowing, low-increasing osteophytes - High-increasing OA features - High osteophytes | 183 (100)  0 (0.0)  0 (0.0)  0 (0.0)  0 (0.0)  0 (0.0) | 1 (0.3)  60 (17.2)  65 (18.6)  112 (32.1)  20 (5.7)  91 (26.1) | 34 (24.8)  30 (21.9)  21 (15.3)  31 (22.6)  3 (2.2)  18 (13.1) | 232 (70.9)  11 (3.4)  41 (12.5)  40 (12.2)  1 (0.3)  2 (0.6) | 0 (0.0)  123 (33.1)  109 (29.3)  1 (0.3)  49 (13.2)  90 (24.2) | 22 (9.3)  11 (4.6)  19 (8.0)  171 (72.2)  1 (0.4)  13 (5.5) | 0.00* |

*ROM: range of motion; CTX-I: C-terminal telopeptide of collagen I; CTX-II: C-terminal telopeptide of type II collagen; C1,2C: collagen of types I and II; COMP: cartilage oligomeric matrix protein; PIIANP: collagen N-propeptide of type IIA; CS846: chondroitin sulphate 846; NTX-I: N-terminal telopeptide of collagen I; OC: osteocalcin; PINP: aminoterminal propeptide of type I procollagen; HA: hyaluronic acid; PIIIANP: N-terminal propeptide of type III procollagen; hsCRP: high-sensitivity C-reactive protein; BSE: erythrocyte sedimentation rate.*

** P-values calculated with chi-square test*

***for missing values, mean of all SOS items was taken (n=6)*

*Median and interquartile range (Q1-Q3) given for continuous variables unless otherwise indicated.*
