# Supplementary material for: Identifying multivariate disease trajectories and potential phenotypes of early knee osteoarthritis in the CHECK cohort
Source: PLoS One. 2023 Jul 14;18(7):e0283717. doi: 10.1371/journal.pone.0283717 (PMC10348540; doi:10.1371/journal.pone.0283717)
Supplement: S3 Table — ROM: range of motion; CTX-I: C-terminal telopeptide of collagen I; CTX-II: C-terminal telopeptide of type II collagen; C1,2C: collagen of types I and II; COMP: cartilage oligomeric matrix protein; PIIANP: collagen N-propeptide of type IIA; CS846: chondroitin sulphate 846; NTX-I: N-terminal telopeptide of collagen I; OC: osteocalcin; PINP: aminoterminal propeptide of type I procollagen; HA: hyaluronic acid; PIIIANP: N-terminal propeptide of type III procollagen; hsCRP: high-sensitivity C-reactive protein; BSE: erythrocyte sedimentation rate. * P-values calculated with chi-square test. (DOCX) [file pone.0283717.s003.docx]

Supplementary Table 3: Descriptive statistics of combined scenario with WOMAC, PF OA features and gender

| **Characteristics** | **Cluster 1** | **Cluster 2** | **Cluster 3** | **Cluster 4** | **Cluster 5** | **Cluster 6** | **Cluster 7** | **Cluster 8** | **P-value** |  |
| --- | --- | --- | --- | --- | --- | --- | --- | --- | --- | --- |
|  | Premeno-pausal females with low and moderate WOMAC pain and function | Males with low WOMAC pain and function | Males with moderate WOMAC pain and function | Postmeno-pausal females with low WOMAC pain and function | Postmeno-pausal females with moderate WOMAC pain and function and low joint space narrowing | Postmeno-pausal females with moderate WOMAC pain and function and varying PF OA characteristics | Postmeno-pausal females with high WOMAC pain and function | Postmeno-pausal females with increasing WOMAC pain and function |  |  |
| n=1605 | n=253 (16%) | n=106 (6%) | n=226 (14%) | n=334 (21%) | n=159 (10%) | n=351 (22%) | n=98 (6%) | n=78 (5%) |  |  |
| Age, years | 49.0 (48.0-52.0) | 57.0 (52.0-62.0) | 56.0 (52.0-61.0) | 58.0 (55.0-60.0) | 57.0 (53.0-61.0) | 57.0 (54.0-61.0) | 57.0 (53.0-61.0) | 56.0 (52.0-61.0) | 0.00 |  |
| Female, n (%) | 253 (100.0) | 0 (0.0) | 26 (11.5) | 334 (100.0) | 159 (100.0) | 338 (96.3) | 97 (99.0) | 56 (71.8) | 0.00* |  |
| Body Mass Index, kg/m^2^ | 25.0 (23.0-28.0) | 25.0 (23.0-27.0) | 26.0 (24.0-29.0) | 26.0 (24.0-29.0) | 25.0 (23.0-28.0) | 25.0 (23.0-28.0) | 25.0 (23.0-28.3) | 27.0 (22.8-29.0) | 0.00 |  |
| Highest completed education level, n (%)   - No school/primary school - Basic vocational education - Secondary education - Secondary vocational education - Higher and university preparatory education - Higher professional education - University | 2 (0.8)  19 (7.6)  50 (20.1)  64 (25.7)  31 (12.4)  73 (29.3)  10 (4.0) | 0 (0.0)  15 (14.2)  12 (11.3)  29 (27.4)  11 (10.4)  31 (29.2)  8 (7.5) | 11 (5.1)  35 (16.1)  36 (16.6)  55 (25.3)  10 (4.6)  52 (24.0)  18 (8.3) | 4 (1.3)  65 (20.4)  97 (30.5)  41 (12.9)  17 (5.3)  65 (20.4)  29 (9.1) | 3 (1.9)  34 (21.9)  50 (32.3)  31 (20.0)  9 (5.8)  19 (12.3)  9 (5.8) | 13 (3.8)  64 (18.7)  109 (31.9)  61 (17.8)  27 (7.9)  44 (12.9)  24 (7.0) | 5 (5.2)  11 (11.5)  29 (30.2)  14 (14.6)  9 (9.4)  24 (25.0)  4 (4.2) | 2 (2.6)  17 (21.8)  20 (25.6)  18 (23.1)  4 (5.1)  15 (19.2)  2 (2.6) | 0.00* |  |
| Smoking, n (%)   - Yes, every day - Yes, occasionally - No, but used to every day - No, but used to occasionally - No, never have | 24 (9.6)  14 (5.6)  102 (41.0)  39 (15.7)  70 (28.1) | 14 (13.2)  7 (6.6)  29 (27.4)  28 (26.4)  28 (26.4) | 29 (13.5)  18 (8.4)  96 (44.7)  37 (17.2)  35 (16.3) | 24 (7.5)  12 (3.7)  94 (29.2)  84 (26.1)  108 (33.5) | 14 (9.0)  7 (4.5)  39 (25.0)  33 (21.2)  63 (40.4) | 30 (8.7)  19 (5.5)  105 (30.4)  77 (22.3)  114 (33.0) | 2 (2.0)  0 (0.0)  34 (34.7)  24 (24.5)  38 (38.8) | 4 (5.1)  2 (2.6)  21 (26.9)  21 (26.9)  30 (38.5) | 0.00* |  |
| Number of comorbidities | 1.0 (0.0-2.0) | 1.0 (0.0-2.0) | 1.0 (0.0-2.0) | 1.0 (1.0-2.0) | 1.0 (1.0-2.0) | 1.0 (0.0-2.0) | 1.0 (1.0-2.0) | 1.0 (1.0-3.0) | 0.01 |  |
| Social Support Scale (SOS) total score** | 14.0 (12.0-18.0) | 16.0 (12.0-19.0) | 16.0 (12.0-20.0) | 15.5 (12.0-21.0) | 16.0 (12.0-24.0) | 15.0 (12.0-23.0) | 15.5 (12.0-22.0) | 16.5 (13.0-24.0) | 0.02 |  |
| Pain Coping Inventory transformation | 2.0 (1.8-2.5) | 1.8 (1.3-2.3) | 2.0 (1.5-2.5) | 2.3 (1.8-2.8) | 2.0 (1.5-2.5) | 2.0 (1.8-2.5) | 2.1 (1.8-2.8) | 2.3 (2.0-2.8) | 0.00 |  |
| Pain Coping Inventory distraction | 2.2 (1.8-2.6) | 1.8 (1.4-2.4) | 2.0 (1.4-2.4) | 2.2 (1.8-2.7) | 2.4 (1.8-2.8) | 2.2 (1.8-2.8) | 2.2 (1.8-2.8) | 2.4 (1.8-2.7) | 0.00 |  |
| Pain Coping Inventory reducing demands | 2.0 (1.7-2.3) | 1.7 (1.3-2.0) | 2.0 (1.7-2.3) | 2.0 (1.7-2.3) | 2.0 (1.7-2.3) | 2.0 (1.7-2.3) | 2.0 (1.7-2.7) | 2.0 (1.7-2.7) | 0.01 |  |
| Pain Coping Inventory retreating | 1.6 (1.1-1.9) | 1.3 (1.1-1.7) | 1.3 (1.1-1.7) | 1.6 (1.1-2.0) | 1.4 (1.1-1.9) | 1.4 (1.1-1.9) | 1.5 (1.1-1.9) | 1.6 (1.1-1.9) | 0.00 |  |
| Pain Coping Inventory worrying | 1.4 (1.2-1.8) | 1.4 (1.4-1.7) | 1.4 (1.2-1.7) | 1.4 (1.2-1.8) | 1.6 (1.3-1.8) | 1.6 (1.2-1.9) | 1.6 (1.2-1.8) | 1.6 (1.2-2.0) | 0.01 |  |
| Pain Coping Inventory resting | 1.8 (1.6-2.2) | 1.6 (1.4-2.0) | 1.8 (1.4-2.0) | 1.8 (1.4-2.2) | 1.8 (1.4-2.1) | 1.8 (1.4-2.2) | 1.8 (1.5-2.4) | 1.8 (1.6-2.0) | 0.00 |  |
| Knee flexion active ROM, degrees | 137.0 (130.0-140.0) | 140.0 (132.0-143.0) | 135.0 (130.0-141.0) | 135.0 (130.0-141.8) | 135.0 (130.0-140.0) | 135.0 (130.0-140.0) | 135.0 (130.0-140.0) | 135.0 (130.0-140.0) | 0.12 |  |
| Knee extension active ROM, degrees | 3.0 (0.0-5.0) | 2.0 (0.0-5.0) | 0.0 (0.0-5.0) | 2.0 (0.0-5.0) | 2.0 (0.0-5.0) | 2.0 (0.0-5.0) | 1.0 (0.0-5.0) | 4.0 (0.0-5.0) | 0.08 |  |
| Hip endorotation active ROM, degrees | 30.0 (25.0-37.0) | 25.5 (20.0-31.3) | 28.0 (20.0-35.0) | 30.0 (25.0-36.0) | 30.0 (26.0-38.0) | 30.0 (25.0-36.0) | 30.0 (25.0-38.0) | 29.5 (21.5-35.0) | 0.00 |  |
| Hip exorotation active ROM, degrees | 30.0 (23.0-40.0) | 30.0 (21.0-35.0) | 29.5 (24.0-35.0) | 27.0 (21.0-35.0) | 28.5 (21.8-33.0) | 29.0 (22.0-35.0) | 30.0 (25.0-35.0) | 29.5 (24.5-35.0) | 0.20 |  |
| Hip flexion active ROM, degrees | 120.0 (110.0-128.0) | 120.0 (110.0-127.3) | 117.0 (110.0-121.5) | 120.0 (110.0-125.0) | 120.0 (110.0-126.0) | 120.0 (112.0-129.0) | 120.0 (110.0-125.0) | 116.0 (110.0-125.0) | 0.01 |  |
| Hip adduction active ROM, degrees | 20.0 (15.0-25.0) | 20.0 (15.0-25.0) | 20.0 (15.0-24.0) | 20.0 (15.0-25.0) | 20.0 (15.0-25.0) | 20.0 (15.0-25.3) | 22.0 (16.0-29.5) | 19.0 (14.0-20.0) | 0.06 |  |
| Hip abduction active ROM, degrees | 31.0 (25.0-40.0) | 33.0 (23.0-40.0) | 31.0 (25.0-40.0) | 30.0 (25.3-39.5) | 31.0 (25.0-37.0) | 30.0 (25.0-40.0) | 31.0 (25.0-38.3) | 30.0 (25.3-40.0) | 0.65 |  |
| Kellgren & Lawrence grade, n (%)   - Grade 0 - Grade 1 - Grade 2 - Grade 3 | 166 (68.0)  58 (23.8)  20 (8.2)  0 (0.0) | 68 (66.0)  31 (30.1)  4 (3.9)  0 (0.0) | 138 (64.2)  54 (25.1)  22 (10.2)  1 (0.5) | 175 (55.9)  101 (32.20  37 (11.8)  0 (0.0) | 93 (60.4)  44 (28.6)  17 (11.0)  0 (0.0) | 197 (58.5)  93 (27.6)  47 (13.9)  0 (0.0) | 58 (60.4)  30 (31.3)  8 (8.3)  0 (0.0) | 45 (60.0)  22 (29.3)  8 (10.7)  0 (0.0) | 0.22* |  |
| Pain or stiffness in hip and/or groin and/or upper part of the upper leg, n (%) | 100 (40.2) | 33 (31.1) | 81 (37.2) | 137 (42.5) | 56 (35.7) | 147 (42.4) | 38 (38.8) | 36 (46.2) | 0.30* |  |
| Pain or stiffness in knee, n (%) | 143 (57.4) | 55 (51.9) | 139 (63.8) | 188 (58.4) | 84 (53.5) | 216 (62.2) | 55 (56.1) | 45 (57.7) | 0.30* |  |
| pLeptin, ng/ml | 14.3 (8.2-24.1) | 3.8 (2.5-6.0) | 5.7 (3.2-8.7) | 14.8 (8.8-26.0) | 13.5 (8.5-23.5) | 13.7 (8.3-27.2) | 15.1 (7.8-26.1) | 9.7 (5.4-26.0) | 0.00 |  |
| pAdiponectin, ug/ml | 9.1 (7.0-13.6) | 7.2 (5.5-9.4) | 7.5 (5.7-9.8) | 11.7 (8.7-15.9) | 11.5 (8.0-15.1) | 10.7 (7.9-15.4) | 12.1 (8.4-18.1) | 10.3 (7.1-15.9) | 0.00 |  |
| pResistin, ng/ml | 3.7 (3.2-4.5) | 3.4 (2.9-4.1) | 3.4 (3.0-4.2) | 3.6 (3.0-4.4) | 3.4 (2.9-4.2) | 3.5 (2.9-4.1) | 3.5 (2.9-4.2) | 3.8 (2.5-4.5) | 0.02 |  |
| uCTX-I, ug/mmol | 114.3 (77.5-173.3) | 113.3 (79.8-181.7) | 110.5 (75.2-148.4) | 187.4 (125.2-255.4) | 204.1 (146.8-265.1) | 184.7 (127.2-255.4) | 180.9 (120.8-240.8) | 143.2 (116.6-219.8) | 0.00 |  |
| uNTX-I, nM BCE/mmol | 31.8 (23.8-42.3) | 27.3 (22.4-42.4) | 29.9 (22.8-38.5) | 41.7 (32.4-56.0) | 46.4 (34.2-57.9) | 41.9 (31.7-57.3) | 40.8 (32.9-51.0) | 38.9 (30.6-54.7) | 0.00 |  |
| sPINP, ug/ml | 34.9 (26.3-47.8) | 35.9 (27.2-45.0) | 37.7 (31.4-47.9) | 46.6 (34.1-62.4) | 49.0 (39.7-64.6) | 46.9 (36.2-57.2) | 45.9 (33.5-56.3) | 39.8 (33.6-49.2) | 0.00 |  |
| sOC | 10.9 (8.3-14.4) | 11.9 (10.2-14.0) | 12.0 (10.5-13.6) | 13.8 (11.4-17.4) | 15.5 (12.1-19.4) | 14.4 (11.7-18.3) | 14.3 (10.3-17.0) | 12.5 (10.4-16.5) | 0.00 |  |
| sC1,2C | 0.18 (0.14-0.23) | 0.16 (0.14-0.19) | 0.16 (0.13-0.19) | 0.19 (0.15-0.23) | 0.18 (0.14-0.21) | 0.18 (0.14-0.24) | 0.17 (0.13-0.22) | 0.14 (0.12-0.19) | 0.00 |  |
| uCTX-II, ng/mmol | 148.5 (107.8-204.2) | 149.1 (114.5-192.3) | 151.2 (113.9-209.8) | 230.3 (164.8-319.2) | 240.6 (168.1-319.8) | 228.8 (151.1-322.0) | 174.7 (129.8-304.8) | 219.7 (165.9-260.4) | 0.00 |  |
| sCS846 | 67.0 (51.4-80.3) | 74.3 (58.0-86.8) | 69.7 (56.8-92.5) | 72.1 (56.1-95.7) | 68.6 (52.4-90.1) | 70.9 (53.7-89.4) | 66.9 (54.6-85.6) | 72.1 (55.2-96.1) | 0.09 |  |
| sCOMP, μg/ml | 7.5 (6.4-9.1) | 9.2 (7.8-10.4) | 9.2 (8.1-10.6) | 8.3 (7.2-10.0) | 8.9 (7.6-10.4) | 8.6 (7.2-10.1) | 8.3 (6.9-9.3) | 8.5 (7.6-9.5) | 0.00 |  |
| sPIIANP | 1280.0 (1000.8-1596.0) | 1391.6 (1048.9-1654.6) | 1130.2 (998.2-1636.6) | 1388.3 (1103.0-1812.5) | 1491.6 (1141.3-1826.2) | 1448.9 (1108.0-1887.8) | 1419.5 (1114.7-1819.2) | 1378.4 (1157.5-1743.1) | 0.00 |  |
| sHA | 17.8 (10.5-26.0) | 31.1 (18.7-56.0) | 32.8 (19.7-44.4) | 31.0 (20.3-50.7) | 29.0 (19.1-41.3) | 27.9 (18.6-46.7) | 26.9 (14.9-44.2) | 30.8 (17.6-42.2) | 0.00 |  |
| sPIIIANP | 4.1 (3.5-4.8) | 3.9 (3.5-4.4) | 4.4 (3.6-5.2) | 4.2 (3.6-5.0) | 4.0 (3.4-4.8) | 4.1 (3.6-4.7) | 4.1 (3.3-5.1) | 4.0 (3.4-4.9) | 0.03 |  |
| hsCRP | 1.5 (0.7-3.5) | 1.0 (0.6-2.0) | 1.5 (0.7-3.2) | 1.6 (0.8-3.4) | 1.5 (0.7-3.4) | 1.5 (0.6-3.3) | 1.1 (0.5-2.3) | 1.1 (0.7-4.0) | 0.08 |  |
| BSE | 7.0 (5.0-12.0) | 5.0 (2.0-6.3) | 5.0 (3.8-8.0) | 9.0 (6.0-14.3) | 10.0 (6.0-16.0) | 9.0 (5.0-15.0) | 8.0 (4.0-15.0) | 9.5 (5.0-14.0) | 0.00 |  |
| WOMAC pain scale standardized (0-100) | 20.0 (10.0-35.0) | 20.0 (10.0-31.3) | 20.0 (10.0-30.0) | 25.0 (15.0-40.0) | 25.0 (10.0-35.0) | 25.0 (15.0-35.0) | 20.0 (13.8-35.0) | 25.0 (10.0-40.0) | 0.00 |  |
| WOMAC physical functioning scale standardized (0-100) | 19.1 (7.4-33.8) | 16.9 (5.9-29.4) | 14.7 (7.4-27.9) | 23.5 (11.8-37.9) | 22.1 (11.8-35.3) | 22.1 (10.3-35.3) | 16.2 (8.8-30.1) | 26.5 (13.2-36.8) | 0.00 |  |
| WOMAC stiffness scale standardized (0-100) | 25.0 (12.5-50.0) | 25.0 (12.5-37.5) | 25.0 (12.5-50.0) | 37.5 (25.0-50.0) | 25.0 (25.0-50.0) | 37.5 (25.0-50.0) | 37.5 (25.0-50.0) | 37.5 (25.0-50.0) | 0.00 |  |
| Lateral Osteophytes: mean of femur and tibia area, mm^2^ | 1.3 (0.0-2.5) | 1.5 (0.5-3.2) | 1.9 (0.5-3.8) | 1.3 (0.0-3.1) | 1.3 (0.0-2.5) | 1.1 (0.0-2.3) | 1.4 (0.0-3.1) | 0.8 (0.0-2.4) | 0.00 |  |
| Medial Osteophytes: mean of femur and tibia area, mm^2^ | 0.5 (0.0-1.5) | 0.7 (0.0-1.9) | 0.9 (0.0-2.1) | 0.9 (0.0-2.0) | 0.6 (0.0-2.1) | 0.5 (0.0-1.8) | 0.8 (0.0-1.6) | 0.0 (0.0-1.4) | 0.11 |  |
| Lateral Joint Space Width, mm | 5.8 (5.1-6.6) | 6.7 (6.0-7.5) | 6.6 (5.8-7.6) | 5.8 (5.0-7.0) | 5.7 (4.9-6.7) | 5.6 (4.8-6.5) | 5.8 (5.0-6.8) | 5.9 (5.1-6.9) | 0.00 |  |
| Medial Joint Space Width, mm | 4.5 (4.1-5.2) | 5.4 (4.8-6.0) | 5.3 (4.7-5.8) | 4.6 (3.9-5.0) | 4.4 (4.0-5.0) | 4.5 (3.9-5.1) | 4.6 (4.1-5.0) | 4.5 (3.9-5.2) | 0.00 |  |
| Lateral Bone Density: mean of femur and tibia area, mm Al eq | 23.5 (20.2-27.3) | 26.2 (23.7-30.6) | 27.6 (24.5-32.1) | 23.0 (20.6-27.4) | 22.8 (19.3-30.0) | 22.2 (18.8-27.4) | 23.1 (20.3-26.7) | 23.8 (20.6-28.1) | 0.00 |  |
| Medial Bone Density: mean of femur and tibia area, mm Al eq | 23.2 (19.5-28.7) | 25.7 (21.9-29.5) | 27.2 (23.2-33.4) | 22.4 (19.8-28.4) | 23.9 (18.8-30.6) | 22.1 (18.2-29.1) | 23.5 (20.2-27.3) | 23.0 (19.5-29.3) | 0.00 |  |
| Knee sky patellofemoral sclerosis score, n (%)   - 0 - 1 - 2 - 3 | 217 (97.7)  4 (1.8)  1 (0.5)  0 (0.0) | 97 (100.0)  0 (0.0)  0 (0.0)  0 (0.0) | 183 (97.3)  5 (2.7)  0 (0.0)  0 (0.0) | 283 (99.6)  0 (0.0)  1 (0.4)  0 (0.0) | 137 (97.9)  1 (0.7)  2 (1.4)  0 (0.0) | 294 (99.3)  2 (0.7)  0 (0.0)  0 (0.0) | 80 (97.6)  2 (2.4)  0 (0.0)  0 (0.0) | 70 (100.0)  0 (0.0)  0 (0.0)  0 (0.0) | 0.08 |  |
| Knee sky patellofemoral narrowing, n (%)   - 0 - 1 - 2 - 3 | 208 (86.0)  26 (10.7)  5 (2.1)  3 (1.2) | 92 (92.0)  4 (4.0)  4 (4.0)  0 (0.0) | 186 (87.3)  22 (10.3)  5 (2.3)  0 (0.0) | 266 (86.1)  40 (12.9)  3 (1.0)  0 (0.0) | 133 (87.5)  16 (10.5)  3 (2.0)  0 (0.0) | 274 (83.3)  48 (14.6)  5 (1.5)  2 (0.6) | 81 (88.0)  8 (8.7)  2 (2.2)  1 (1.1) | 57 (78.1)  14 (19.2)  2 (2.7)  0 (0.0) | 0.15 |  |
| Knee sky patellofemoral osteophytes, n (%)   - 0 - 1 - 2 - 3 | 136 (56.4)  78 (32.4)  23 (9.5)  4 (1.7) | 45 (44.6)  49 (48.5)  7 (6.9)  0 (0.0) | 103 (48.6)  80 (37.7)  27 (12.7)  2 (0.9) | 134 (44.7)  125 (41.7)  40 (13.3)  1 (0.3) | 71 (47.7)  58 (38.9)  18 (12.1)  2 (1.3) | 153 (47.2)  124 (38.3)  40 (12.3)  7 (2.2) | 42 (46.7)  38 (42.2)  9 (10.0)  1 (1.1) | 30 (41.1)  38 (52.1)  5 (6.8)  0 (0.0) | 0.16 |  |
| Knee replacement, n (%) | 4 (1.6) | 2 (1.9) | 4 (1.8) | 11 (3.3) | 3 (1.9) | 18 (5.1) | 0 (0.0) | 2 (2.6) | 0.05 |  |
| Hip replacement, n (%) | 9 (3.6) | 8 (7.6) | 10 (4.4) | 8 (2.4) | 11 (6.9) | 20 (5.7) | 3 (3.1) | 7 (9.0) | 0.05 |  |
| Membership to WOMAC pain trajectories, n (%)   - Moderate - Increasing - Decreasing - High - Low | 129 (51.0)  10 (4.0)  8 (3.2)  5 (2.0)  101 (39.9) | 4 (3.8)  2 (1.9)  0 (0.0)  0 (0.0)  100 (94.3) | 116 (51.3)  6 (2.7)  50 (22.1)  28 (12.4)  26 (11.5) | 3 (0.9)  4 (1.2)  4 (1.2)  0 (0.0)  323 (96.7) | 118 (74.2)  9 (5.7)  3 (1.9)  8 (5.0)  21 (13.2) | 246 (70.1)  16 (4.6)  26 (7.4)  16 (4.6)  47 (13.4) | 3 (3.1)  2 (2.0)  0 (0.0)  93 (94.9)  0 (0.0) | 3 (3.8)  72 (92.3)  2 (2.6)  1 (1.3)  0 (0.0) | 0.00* |  |
| Membership to WOMAC function trajectories, n (%)   - Moderate - Increasing - Decreasing - High - Low | 104 (41.1)  0 (0.0)  33 (13.0)  16 (6.3)  100 (39.5) | 18 (17.0)  4 (1.8)  0 (0.0)  0 (0.0)  88 (83.0) | 116 (51.3)  4 (1.8)  62 (27.4)  24 (10.6)  20 (8.8) | 44 (13.2)  0 (0.0)  0 (0.0)  0 (0.0)  290 (86.8) | 102 (64.2)  5 (3.1)  25 (15.7)  6 (3.8)  21 (13.2) | 221 (63.0)  6 (1.7)  71 (20.2)  31 (8.8)  22 (6.3) | 8 (8.2)  0 (0.0)  11 (11.2)  79 (80.6)  0 (0.0) | 15 (19.2)  56 (71.8)  0 (0.0)  4 (5.1)  3 (3.8) | 0.00* |  |
| Membership to WOMAC stiffness trajectories, n (%)   - Moderate - Increasing - Decreasing - High - Low | 97 (38.3)  32 (12.6)  29 (11.5)  41 (16.2)  54 (21.3) | 40 (37.7)  16 (15.1)  16 (15.1)  15 (14.2)  19 (17.9) | 78 (34.5)  26 (11.5)  20 (8.8)  41 (18.1)  61 (27.0) | 137 (41.0)  27 (8.1)  40 (12.0)  51 (15.3)  79 (23.7) | 50 (31.4)  21 (13.2)  15 (9.4)  27 (17.0)  46 (28.9) | 141 (40.2)  38 (10.8)  46 (13.1)  53 (15.1)  73 (20.8) | 45 (45.9)  8 (8.2)  10 (10.2)  15 (15.3)  20 (20.4) | 40 (51.3)  6 (7.7)  0 (0.0)  7 (9.0)  25 (32.1) | 0.05* |  |
| Membership to TF OA trajectories, n (%)   - Low osteophytes, stable bone density - Increasing bone density - Low bone density - Increasing lateral osteophytes, average bone density - Increasing (lateral and medial) osteophytes, low medial JSW, high lateral JSW, increasing bone density - Moderate high bone density - Increasing high bone density - Slightly increasing bone density | 59 (23.3)  42 (16.6)  23 (9.1)  18 (7.1)  15 (5.9)  58 (22.9)  9 (3.9)  29 (11.5) | 26 (24.5)  13 (12.3)  10 (9.4)  10 (9.4)  6 (5.7)  19 (17.9)  11 (10.4)  11 (10.4) | 48 (21.2)  31 (13.7)  26 (11.5)  16 (7.1)  8 (3.5)  61 (27.0)  12 (5.3)  24 (10.6) | 78 (23.4)  50 (15.0)  40 (12.0)  29 (8.7)  13 (3.9)  63 (18.9)  22 (6.6)  39 (11.7) | 26 (16.4)  30 (18.9)  17 (10.7)  14 (8.8)  1 (0.6)  38 (23.9)  10 (6.3)  23 (14.5) | 75 (21.4)  58 (16.5)  47 (13.4)  28 (8.0)  8 (2.3)  83 (23.6)  18 (5.1)  34 (9.7) | 20 (20.4)  17 (17.3)  9 (9.2)  3 (3.1)  6 (6.1)  19 (19.4)  8 (8.2)  16 (16.3) | 13 (16.7)  11 (14.1)  12 (15.4)  6 (7.7)  1 (1.3)  19 (24.4)  1 (1.3)  15 (19.2) | 0.29* |  |
| Membership to PF OA trajectories, n (%)   - Low joint space narrowing, moderate osteophytes - Moderate-increasing OA features - Low OA features - Low joint space narrowing, low-increasing osteophytes - High-increasing OA features - High osteophytes | 80 (31.6)  42 (16.6)  36 (14.2)  56 (22.1)  3 (1.2)  36 (14.2) | 34 (32.1)  9 (8.5)  11 (10.4)  34 (32.1)  0 (0.0)  18 (17.0) | 78 (34.5)  31 (13.7)  51 (22.6)  42 (18.6)  5 (2.2)  19 (8.4) | 86 (25.7)  40 (12.0)  42 (12.6)  94 (28.1)  15 (4.5)  57 (17.1) | 154 (96.6)  0 (0.0)  3 (1.9)  1 (0.6)  1 (0.6)  0 (0.0) | 0 (0.0)  79 (22.5)  92 (26.2)  75 (21.4)  46 (13.1)  59 (16.8) | 22 (22.4)  25 (25.5)  3 (3.1)  30 (30.6)  3 (3.1)  15 (15.3) | 18 (23.1)  9 (11.5)  17 (21.8)  23 (29.5)  1 (1.3)  10 (12.8) | 0.00* |  |
| *ROM: range of motion; CTX-I: C-terminal telopeptide of collagen I; CTX-II: C-terminal telopeptide of type II collagen; C1,2C: collagen of types I and II; COMP: cartilage oligomeric matrix protein; PIIANP: collagen N-propeptide of type IIA; CS846: chondroitin sulphate 846; NTX-I: N-terminal telopeptide of collagen I; OC: osteocalcin; PINP: aminoterminal propeptide of type I procollagen; HA: hyaluronic acid; PIIIANP: N-terminal propeptide of type III procollagen; hsCRP: high-sensitivity C-reactive protein; BSE: erythrocyte sedimentation rate.* | | | | | | | | | | |
| ** P-values calculated with chi-square test* | | | | | | | | | | |
